# Up to your NEK2 in CIN

**DOI:** 10.18632/oncotarget.27918

**Published:** 2021-04-13

**Authors:** Darcie D. Seachrist, Lindsey J. Anstine, Ruth A. Keri

**Keywords:** LIN9, NEK2, chromosomal instability, taxane resistance, breast cancer

Cancers are, first and foremost, a disease of unrestrained proliferation. Although other cancer hallmarks aid and abet advanced disease, the most effective therapies are those that disrupt proliferation [1]. Selective drivers of tumor growth have been identified for several cancer types, and targeted therapies to these factors have significantly extended patient outcomes [2–5]. For the majority of cancers, non-selective chemotherapies remain the most effective for achieving regression of both primary and metastatic lesions. Despite their efficacy at targeting proliferating cells, chemotherapeutic drugs typically have a narrow therapeutic window due to off-target toxicity, and many patients develop resistance, underscoring the need for more effective and safer options for patients. With recent discoveries, it is becoming increasingly apparent that the drivers of proliferation itself may function in a tissue-specific manner [6]. As a consequence of enforcing cell cycle progression, these drivers often induce mitotic errors and aneuploidy [7]. The identification and therapeutic targeting of tumor-selective drivers crucial for tumor cell proliferation in the face of unstable aneuploidy could reverse disease progression, overcome resistance, and reduce widespread toxicity in many cancer types.

Triple-negative breast cancers (TNBC) display high levels of intra- and inter-tumoral heterogeneity, making the identification of universal drivers challenging for this cancer class [8]. As a result, few targeted therapies are FDA approved for TNBC, and patients are most commonly treated with taxanes, alone or in combination with other cytotoxic agents such as anthracyclines, as standard of care. Paclitaxel, the most widely used taxane, stabilizes microtubule spindles during mitosis, causing mitotic errors. These genomic insults result in mitotic catastrophe, cell death, and complete tumor regression for roughly half of TNBC patients, extending their overall survival. However, outcomes are less favorable for those patients with residual disease [9]. Emerging data demonstrate that TNBCs harbor increased levels of unstable aneuploidy, mitotic errors, and chromosomal instability (CIN), and together these are responsible for the heterogeneity and therapeutic resistance observed in this disease [10–12]. While low/moderate level CIN promotes cancer cell fitness, allowing cells to adapt and survive chromosomal insults and environmental stress, the excessive rates of CIN induced by taxanes causes excessive, unrecoverable damage [12]. Cells that become resistant to taxanes have often adapted to elevated CIN. Discovering factors that tumor-specifically promote CIN and can be leveraged to further elevate it to intolerable levels may provide therapeutic targets for rational drug design in TNBC as well as other cancers.

**Figure 1:**
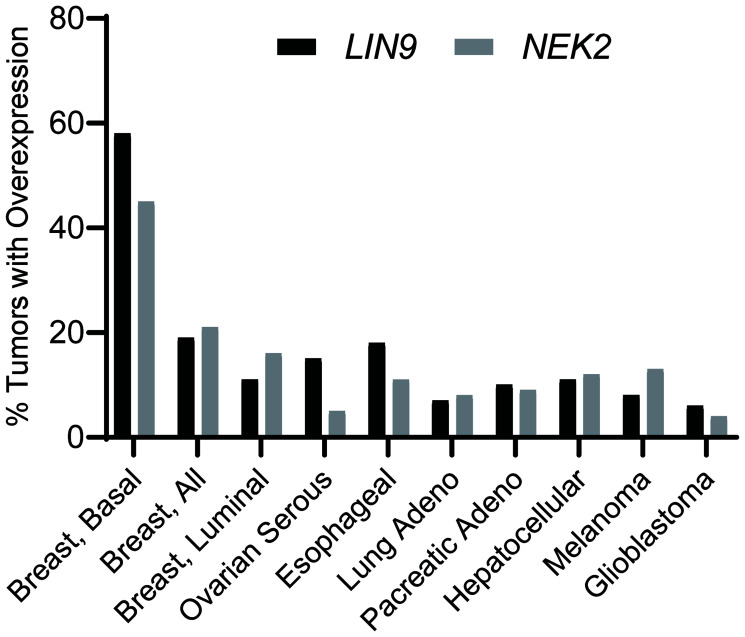
*LIN9* and *NEK2* overexpression in various human cancers. Percent of tumors with overexpression of either *LIN9* or *NEK2* across cancer types. Graph generated using RNA-Seq data generated by the TCGA Research Network [21] (http://www.cancer.gov/tcga), archived in cBioPortal and expressed relative to diploid samples; Z-score threshold ≥ 2.0.

We recently identified the tissue-selective upregulation of the LIN9/NEK2 pathway in TNBC that we propose can be leveraged to ameliorate taxane resistant disease [13–15]. LIN9 is the scaffolding component of the MUVB complex that cooperates with transcriptional modulators, such as FOXM1 and B-MYB, to control the expression of cell cycle genes and proper mitotic progression. LIN9 expression is selectively upregulated in TNBC compared to other breast cancer subtypes and overexpressed in the majority of TNBC. Notably, elevated *LIN9* expression is relatively unique to TNBC as a class, as other highly proliferative and highly aggressive malignancies such as glioblastoma and ovarian carcinoma do not show this same trend (Figure 1) [16, 17]. Silencing LIN9 expression in TNBC cells resulted in multiple mitotic errors that manifested as nuclear abnormalities such as multi-nucleation, micro-nucleation, and dysmorphic nuclei as well as amplified and aberrant centrosomes. Although these events increased cell death in TNBC, the effect of LIN9 silencing on cell viability was even more profound in the context of taxane resistance. Indeed, paclitaxel resistant cells displayed a further upregulation of LIN9 expression compared to their taxane sensitive counterparts. Moreover, silencing LIN9 expression amplified the mitotic errors associated with resistance and restored taxane sensitivity. These results indicated that the elevated LIN9 expression observed with taxane resistance enables tolerance of chromosomal instability and promotes cell viability. This also suggested that selective LIN9 inhibitors might extend survival in patients with taxane resistant disease. Although compelling, developing inhibitors to LIN9 is considered challenging as it lacks unique and readily defined binding pockets for drug accessibility, a common feature of transcription and protein-interacting factors. To overcome this challenge, we elected to identify downstream effectors of LIN9 that were critical for maintaining the chromosomal instability observed in taxane resistant cells and could provide a viable therapeutic targeting strategy. To accomplish this, we intersected publicly available ‘omics data with the goal of selecting druggable candidates that met the following conditions: 1) mRNA expression must be highly correlated with *LIN9* in TNBC, 2) high expression of the target must be prognostic of worse patient outcomes (similar to *LIN9*), 3) target must be a direct transcriptional target of LIN9, 4) the target should have established functions in maintaining centrosome function, consistent with our observation of centrosome abnormalities, and 5) selective inhibitors must be readily available to inhibit candidate function. The result of this selection criteria yielded one candidate, NIMA-like Kinase 2 (NEK2). A “druggable” enzyme, NEK2 is a multifunctional serine/threonine kinase that governs several steps in mitotic progression, such as centrosome duplication, kinetochore detachment, and the spindle assembly checkpoint [18]. NEK2 also controls microtubule detachment, an essential step in mitosis also targeted by taxanes [19]. Moreover, we found that NEK2 dysregulation causes the accumulation of mitotic errors reminiscent of those occurring with LIN9 silencing, affirming it as a viable candidate to target for taxane resensitization. Indeed, we found that inhibiting NEK2 function, using either genetic or pharmacological approaches, phenocopied the mitotic errors and catastrophic cell death observed with LIN9 silencing. Notably, increased *NEK2* expression is prognostic of worse outcomes for TNBC patients with residual disease following adjuvant taxane/anthracycline treatment, suggesting that NEK2 may be a useful biomarker to predict chemotherapeutic response. Lastly, we found that *NEK2* inhibition induced regression of both paclitaxel sensitive and resistant TNBC tumors in orthotopic xenograft mouse models of TNBC.

This study reinforces the utility of targeting factors that drive mitotic progression and CIN and affirm that mechanisms fostering unstable aneuploidy may be leveraged for therapeutic benefit. More specifically, these data provide foundational support for inhibiting the LIN9/NEK2 pathway to sensitize TNBCs to chemotherapies such as taxanes. *LIN9* and *NEK2* are upregulated in a substantial proportion of TNBC (Figure 1) [16, 17], hence inhibitors of these proteins have the potential to improve outcomes for a large subset of patients with this disease. Although *LIN9* and *NEK2* expression is selectively upregulated in TNBC, NEK2 can also be expressed in a variety of cancers and is prognostic of drug resistance, rapid relapse, and poor patient survival [20]. The absence of *LIN9* upregulation in the majority of these other cancer types suggests that there may be multiple pathways contributing to NEK2 upregulation that are tissue-selective. Identifying alternative pathways that control NEK2 expression may reveal additional therapeutic targets for further drug development that complement current efforts to design selective inhibitors of NEK2 for clinical utility.

## References

[1] Sun J , et al. BMC Syst Biol. 2017; 11:87. 10.1186/s12918-017-0464-7. 28984210 PMC5629554

[2] Slamon DJ , et al. N Engl J Med. 2001; 344:783–92. 10.1056/nejm200103153441101. 11248153

[3] Chapman PB , et al. N Engl J Med. 2011; 364:2507–16. 10.1056/NEJMoa1103782. 21639808 PMC3549296

[4] Druker BJ , et al. N Engl J Med. 2001; 344:1038–42. 10.1056/nejm200104053441402. 11287973

[5] DeFriend DJ , et al. Cancer Res. 1994; 54:408–14. 8275477

[6] Sack LM , et al. Cell. 2018; 173:499–514.e23. 10.1016/j.cell.2018.02.037. 29576454 PMC6643283

[7] Levine MS , et al. Genes Dev. 2018; 32:620–38. 10.1101/gad.314351.118. 29802124 PMC6004076

[8] Bianchini G , et al. Nat Rev Clin Oncol. 2016; 13:674–90. 10.1038/nrclinonc.2016.66. 27184417 PMC5461122

[9] Bagegni NA , et al. PLoS One. 2019; 14:e0222358. 10.1371/journal.pone.0222358. 31536530 PMC6752843

[10] Rieckhoff J , et al. Cancers. 2020; 12:2809. 10.3390/cancers12102809. 33003585 PMC7601067

[11] Bakhoum SF , et al. Cold Spring Harb Perspect Med. 2017; 7:a029611. 10.1101/cshperspect.a029611. 28213433 PMC5453382

[12] Swanton C , et al. Proc Natl Acad Sci U S A. 2009; 106:8671–6. 10.1073/pnas.0811835106. 19458043 PMC2688979

[13] Roberts MS , et al. Cancer Res. 2020; 80:1693–706. 10.1158/0008-5472.CAN-19-3466. 32054769 PMC7165041

[14] Gayle SS , et al. Oncoscience. 2017; 4:128–30. 10.18632/oncoscience.372. 29142903 PMC5672896

[15] Sahni JM , et al. Cancer Res. 2017; 77:5395–408. 10.1158/0008-5472.CAN-17-1571. 28807940 PMC5626629

[16] Cerami E , et al. Cancer Discov. 2012; 2:401–4. 10.1158/2159-8290.Cd-12-0095. 22588877 PMC3956037

[17] Gao J , et al. Sci Signal. 2013; 6:l1. 10.1126/scisignal.2004088. 23550210 PMC4160307

[18] Fang Y , et al. Cell Cycle. 2016; 15:895–907. 10.1080/15384101.2016.1152430. 27019372 PMC4889274

[19] Du J , et al. Oncogene. 2008; 27:4107–14. 10.1038/onc.2008.34. 18297113 PMC7983366

[20] Kokuro T , et al. Anticancer Res. 2019; 39:2251–8. 10.21873/anticanres.13341. 31092416

[21] Cancer Genome Atlas Network. Nature. 2012; 490:61–70. 10.1038/nature11412. 23000897 PMC3465532

